# The College Bill in the House of Lords

**Published:** 1919-05-31

**Authors:** 


### NURSE REGISTRATION.

### THE COLLEGE BILL IN THE HOUSE OF LORDS.

### Second Reading Carried; Majority 3 to 1.

### OFFICIAL VERBATIM REPORT OF DISCUSSION.

### Tuesday, May 27.

**Viscount Goschen:** My Lords, in moving the Second Reading of this Bill for the registration and training of nurses, I do not think I need say much with regard to the general principle of registration, because that was accepted by your Lordships in this House on the passing of the Registration Bill brought forward by Lord Ampthill, and passed without a Division. There is also a Bill before another place which has passed its Second Reading and has gone to a Standing Committee.

Among the great schemes of reconstruction at the present moment there is none, I suppose, more important than that dealing with the health of the people, a Bill for which has lately been passed by your Lordships' House. The Parliamentary Secretary to the Local Government Board in another place said that the success of the new Ministry of Health set up under that Bill would depend very largely upon the instruments by which it was carried out. One of the instruments for carrying out that Bill is the nursing profession. In recent years a great deal of new work has been entrusted to the nursing profession by the Board of Education and by the Local Government Board, and nurses have had new opportunities of teaching hygiene and the laws of health, and I am quite sure that anything which enhances the status and efficiency of nurses must be good for the public at large and for the nation. I venture to think that the fact that they are doing so much public work is another argument in favour of their registration, but such a step, I think, ought to be part of the big scheme which is being set up by the Government at the present moment to deal with the health of the country.

The registration of nurses—that is to say, the setting up of a Register upon which only nurses who have gone through a certain training and reached a certain standard of efficiency can be placed—must be a protection not only to the public but also to the nurses themselves; for the public, if it employs a registered nurse, would know that it is employing a nurse who has reached a standard of efficiency and has certain qualifications; and the nurses themselves will be protected as regards their name, pay, and uniform—a thing which is very important at the present moment—and will achieve a status which the devoted services that nurses have always given thoroughly deserve.

There is one misunderstanding with regard to a system of registration which I should like to dispel. Registration would not prevent any one from nursing, but only those can claim to be put upon the Register who have reached that standard of efficiency and have got those qualifications which the Bill sets up. I may point out that the system of registration has already been put into force in many of our Colonies, and it is, of course, in force with regard to the medical profession and dentists. There are two methods by which a system of registration can be set up. Firstly, there is the method by which sanction for registration can be asked for and an assurance given that when once registration is promoted the work will be carried on. There is a second method, which is, in the first place, to consider the problem of registration, to have some experience of the difficulties, to carry on some experimental work with regard to registration, and then to obtain the sanction of Parliament for a Registration Bill. It is in accordance with the second of those methods that this Bill is introduced.

The College of Nursing, which is promoting this Bill, has already compiled a Register of some 14,000 fully-trained nurses, drawn from practically every training-school in the country and registered in accordance with a definite plan, providing in nearly every instance a three years' training, and it has during its three years of existence had some experience of the setting up of a Provisional Council, and also of a Council which is at the present moment being elected by the nurses themselves. Therefore it has to-day some experience of the problem incidental to registration.

Let me make quite clear to your Lordships the position of the College of Nursing under the Bill. The College started as a purely voluntary body which endeavoured to secure the co-operation of the nurses' training-schools in the country, and it has obtained from them a very large measure of support, and also a large measure of support from the nurses themselves, as can be shown from the number who have joined its Register. But, besides registration, the College of Nursing has other objects in view, such as to make provision for scholarships, for studentships, and for a professional journal, the aim of all this being to promote the efficiency of the nurses and to see that that efficiency is maintained after they are registered, and to give them an opportunity of keeping up a knowledge of their work.

In 1916 the College was incorporated under the Companies Act as a company limited by a guarantee and without share capital. Under this Bill it is now asking to drop the word "Limited," because if it comes under the Bill that would no longer be necessary. Besides, it is already precluded from declaring a dividend or distributing any profits. Sub-section (2) of Clause 1 says that the College of Nursing shall be administered by the General Nursing Council—that is, that the College, with its Register of nurses, and with its funds, which amount to somewhere about £40,000, shall be handed over to the General Nursing Council to be administered by them; but the College is kept in existence under sub-section (3) of Clause 5. The reason for this provision is that the other objects of the College, to which I have called your Lordships' attention and which are so valuable to the nursing profession, may be kept alive. That is, if I may so call it, the basis of the Bill. The College of Nursing is to be handed over to the General Nursing Council, but, so far as its educational side is concerned, it is to be kept alive.

As to the general scheme of the Bill, a General Nursing Council is to be set up to carry out the provisions of the Bill, the exact composition of which Council is to be decided upon by the Privy Council after receiving a scheme from the Provisional Council, but the Bill lays down as essential that there should be proportional representation of various bodies who are interested in the training of nurses and proportional representation of the nurses themselves.

An endeavour has been made in this Bill to secure two objects: one is elasticity, and the other democratic representation. The first object is secured under Clause 4 and Clause 5, sub-section (2). The object of Clause 4 is that if it is desired to institute Supplementary Registers of Nurses—that is to say, registers of nurses who are nursing in a children's hospital, or in a fever hospital, or a municipal hospital—that can be done by the sanction of the

**Nurses' Registration in the House of Lords**—(*cont.*).

Privy Council, and it will not be necessary to introduce an amending Act. In Clause 5, sub-section (2), it is stated that the rules under this Bill must be approved by the Privy Council—thus, I may point out, giving Parliamentary control—and also that a modification may be made in such rules by the Privy Council. Thus, I think, any rigidity is done away with, and fresh situations can be dealt with under the Bill as they arise.

The second object of the Bill is democratic representation, and this, I think, is secured by Clause 5, sub-section (1), paragraph (a), which lays down that two-thirds of the Council shall be elected by the nurses on the General Register. My postbag during the last two days has rather suggested to me that by some occult means I am attempting under this Bill to disfranchise the nurses. I cannot possibly see how this is being done, for under this clause the nurses are free to vote for any one—for a man or a woman, for a doctor, for a matron, for a nurse, or for anybody interested in hospital work. Two-thirds of the Council are to be elected by them. They are free to put upon the Council, as regards these two-thirds, any person whom they think can best serve them and their interest. And this does seem to me to secure to them adequate control and to give them freedom of choice in the selection of their representatives.

Having thus dealt briefly with the basis of the Bill and the general scheme, may I show how this scheme is to be carried out? For the purpose of compiling the first Register of Nurses a Provisional Nursing Council is set up under Clause 7, consisting of thirty-six members, representing the nursing profession, the Poor-Law unions, the hospital training-schools, twelve representatives of the Central Committee for the State Registration of Nurses, which is the Committee that promoted a Bill in another place; and twelve of the Council of the College of Nursing, who are responsible for this Bill. So that it will be seen that under this clause both parties have an equal representation.

This Provisional Council will start with a Register of 14,000 nurses, which is handed over to it by the College of Nursing. Directly it is appointed it has two duties to carry out. The first is to form a scheme regulating the constitution of the General Nursing Council, which it will submit to the Privy Council; and the second is to originate a Register of nurses in training at the moment when the Bill is passed, according to Clause 5, subsection (1) (*d*). It has been thought advisable not to maintain the Provisional Council in existence for a longer time than necessary, but to expedite the establishment of the General Council of Nursing in order to secure at the earliest moment the direct representation of the nurses themselves. It is therefore laid down in the Bill that when the number of nurses on the Register comes to 30,000 an election for the General Council shall take place. There is no magic in the number of 30,000, but as 14,000 nurses are to be handed over on the Register of the College of Nursing, it seemed only fair to wait until that number had been more than equalled before an election should take place for the General Council. Directly the General Council is appointed it will proceed to carry out all the rules set out under Clause 5, which put in detail the principles contained in other clauses.

There are various clauses dealing with the registration of nurses. Clause 3 sets up the Register and admits the registered nurses from the College of Nursing without any fee, for they have paid for 14,000, to be included in the number handed over. Clause 9 provides for the existing nurses who claim to be registered within three years of the passing of the Act, and who are to be admitted to the Register subject to certain provisions prescribed under the rule. Clause 9 defines the qualifications of the nurses who will be put on the Register after the three years of grace have expired. Clause 13 limits the fee to a guinea; and Clauses 14 and 17 define the penalties for pretending to be registered and for obtaining a certificate by false representation. Clause 19 provides an appeal to the High Court for any nurse who is aggrieved at a decision of the General Council to remove her name from the Register

I am afraid that the details of the Bill are somewhat technical, but I have endeavoured to deal with them as briefly as possible. If I may sum up, I would ask your Lordships to support the Bill because it is wide in its scope, in that it has, beyond a scheme of registration, a system for the educational advancement and social betterment of the nurses. As I have said, it has democratic representation, because it is the object of the Bill to substitute a General Council of Nurses, elected as to two-thirds by the nurses themselves as soon as possible and without fixing a definite period for the Provisional Council, and it has this elected Council which will have to make the rules under which the nursing profession will have to live.

There is a Bill for the registration of nurses introduced in another place. I should like to assure your Lordships that neither I nor any of those associated with me have introduced this Bill in any spirit of aggression. The subject is much too important to the country and to the nurses, and it has been much too long delayed that any undue rivalry of parties who are interested in it and who are, after all, aiming at the same goal, although by somewhat different roads, should in any way impede or check its progress. What I hope is that your Lordships will give a Second Reading to this Bill to-day, and that an opportunity may be found for referring both Bills to a joint consideration, either to a Select Committee or by some other means, in order that from such joint consideration there may emerge an agreed Bill which will set up an efficient system of registration that will be satisfactory to the public and to the nurses themselves. I beg to move.

Moved, That the Bill be now read 2a.—(*Viscount Goschen.*)

**Lord Ampthill** had given notice, on the Motion for the Second Reading, to move that the Bill be read 2a this day six months. The noble Lord said: My Lords, it is a matter of great regret to me that in moving the rejection of this Bill I should find myself in opposition to the objects and wishes of many good friends of mine, and in conflict with their opinions. It is only a strong sense of duty which obliges me to take up this position. It was twelve years ago that I took up this question of State Registration of nurses, because I had satisfied myself that such a measure was necessary as a protection to the public, as an act of justice to women generally, and to nurses in particular, and one which was required for the advancement of a noble profession. I cannot abandon those whose cause I have espoused; and, having once put my hand to the plough, I am naturally going to resist the endeavours of those who wish to turn that plough aside from the straight furrow which it has been pursuing for the past twelve years. That is why I have had to turn a deaf ear to those friends who say, "Leave it alone," and who warn me that I am running some risk of incurring displeasure among my friends—but I trust not any animosity.

Let me assure your Lordships that I have no personal or particular interests to serve. For some time past I have ceased to be the manager of a hospital, and the only thing with which I am concerned is that nurses should secure State Registration. So far as I am personally concerned, after four and a-half years in the Army, I feel that it is "anything for a quiet life"; and I can assure your Lordships that it is not for my own amusement that I am here opposing this Motion to-night. My noble friend has spared me the necessity of reminding your Lordships of the need for State Registration of nurses. He has spoken very strongly in favour of that principle, and some of your Lordships who have not had time to consider the matter may wonder why I, who have been for so long an advocate of State Registration, should be opposing this Bill, which calls itself "a Bill for the registration of nurses." It is just because this Bill is not a Registration Bill proper that I am opposing it.

The primary object of this Bill is not the State Registration of nurses. If your Lordships will look at it you will see that the object which is set out in the very forefront of

**Nurses' Registration in the House of Lords**—(*cont.*).

the Bill, and which runs through every one of its clauses, is to secure the incorporation of a private company known as the College of Nursing, Limited, and, of course, to secure for that company financial support under the *ægis* of State authority. I hold that this is not the right thing to do, and, even if it were, this is not the right way to do it. If you wish to give these privileges to this company, if you wish to give them what is in effect a monopoly in the organisation and the union of all trained nurses so that they may control them for their own purposes, let that be clearly declared on any Bill which you accept; let it appear in the Title of the Bill and in the objects and reasons which are given to the public at large.

Many of the promoters of this Bill were a short time ago most determined opponents of State Registration of nurses. If you doubt my word on that point, ask my noble friend Lord Knutsford. I heard that he has been going about saying to the advocates of State Registration, "You would never have got your Bill unless some one had bribed away my matrons." What does he mean by that? He means that those people who were formerly against registration and who are now professing to be in favour of it must have been offered some consideration; that there is some ulterior motive which has appealed to them; that they are not advocating registration now simply and solely for the sake of registration. But if the promoters of this College Bill had as their only object the registration of nurses, surely they would have contented themselves with taking the Bill which is now before the House of Commons, which was before your Lordships' House eleven years ago, which passed through this House without a Division, which later was accepted in principle by the House of Commons—which, in short, is the Bill that holds the field, and has been generally approved in principle. They would have taken that Bill and would have contented themselves with moving Amendments in the House of Commons and later, if necessary, also in your Lordships' House.

My noble friend the mover of this Bill suggests that if your Lordships give it a Second Reading it will be possible to refer it to some Joint Committee, so that both Bills may be considered together. I may tell your Lordships that there have been many conferences between the supporters of this Bill and the supporters of the Bill which is before the House of Commons. Concessions have been made to the supporters of the College of Nursing Bill, and they have lost a splendid opportunity—firstly, by asking too much; and, secondly, by failing on many occasions to stick to agreements which they have made. My noble friend skated very skilfully over thin ice by drawing an entirely fanciful distinction between the two methods by which registration might be established, and he declared that the principle of his Bill was that of the "experimental method." You will see that this "experimental method"—if you have had the time to study the literature which has been liberally showered upon you—consists of methods which cannot be regarded as entirely fair. The College of Nursing gave a promise to nurses which they had no right to give, because it was a promise which they had no power to fulfil. They said, "If you will join our College and pay us one guinea you shall be registered without any further fee." That is what my noble friend calls making an experimental Register. In the first place, I may say that there is no need whatever to make an experimental Register. Once you grant State Registration there will be no difficulty whatever about carrying it out, nor will there be any reluctance on the part of any trained nurse to get her name registered. Experiment, therefore, is entirely unnecessary and beside the point.

The supporters of the College of Nursing Bill have been exceedingly busy. I understand that they have been advocating their Bill not only inside this House but outside, and that their method has been one of confident assertion. I know as a fact that some members of your Lordships' House, who admit that they have not read either Bill, have gone away in consequence of this "lobbying" with the notion that Lord Goschen's Bill is the better. Others, again, with equally imperfect knowledge of the subject, have been seized with the notion that this is a democratic Bill, whereas the Bill before the House of Commons is not democratic. Both of those claims are utterly unfounded, as I shall presently proceed to show your Lordships. But to me this is yet another striking illustration of the fact that one of the best ways of getting on in the political world is by sheer effrontery. I have had no time for doing any of this "lobbying," and I have neither the talent nor the inclination for that kind of work. I have come here relying entirely on the strength of my case, and I rely upon it even though it is not in my power to put it before you as strongly as it ought to be put.

I am going to try to justify my motion for the rejection of this Bill first of all on grounds of expediency; secondly, on grounds of principle; and, thirdly, because the Bill itself is a thoroughly bad Bill. In the matter of expediency, the original Bill for the State Registration of Nurses—the Bill which I had the honour of piloting through this House eleven years ago, which your Lordships accepted without a division, and which, several years later, was approved in principle by the House of Commons—has now made good progress in the House of Commons. It passed second reading with general approval of members of all parties, and it has been through the Standing Committee. Why complicate the issue by introducing a totally different scheme at this stage? Why go out of your way to be at cross-purposes with the other House, especially at a time like this, when it is all-essential that both Houses should be in agreement and when there is such an enormous number of important political measures which you have to discuss and bring into law?

Why delay registration? With the exception of my noble friend on the right, Lord Knutsford, everybody is agreed that we have to have registration. Why delay when it is not necessary to do so? The only effect of giving a second reading of this Bill is that there will be an indefinite amount of delay. You are not going to get the House of Commons to agree to this Bill. They have already approved of the other, and you cannot reconcile these two Bills because they are totally and fundamentally different in principle. It may possibly be a better plan to give this power to the College of Nursing, but the other plan holds the field. Your Lordships are committed to it. You have accepted it in principle. The other House of Parliament is committed to it. Therefore, it is short-sighted, a waste of time, and utterly futile to go back and start afresh on a new scheme in regard to which it would certainly take years to secure the agreement of all the parties concerned. Another reason of expediency is that, if your Lordships accept this Bill or if you delay registration, you will force nurses into trade unions. This is not an idle threat. It is what is already happening. You have seen it in the case of the Asylum Workers' Association, and if you force nurses to form trade unions in order to secure that which they regard, and rightly regard, as a measure of justice and a right to them, you will simply throw them into the arms of the Labour Party. Is that a desirable thing to do at the present time?

This is a question which your Lordships can settle by agreement with that which the other House has already done. This House stands high in the estimation of the associations of nurses, and you can get the credit for granting them that for which they have struggled so long, so bravely, and so persistently. Why throw that away? I think it would be the greatest possible mistake. Why, in these days when you have not too much credit in the eyes of the general public, forfeit the good opinion which you hold and which you have the power to retain? Another reason of expediency is that your Lordships would be simply wasting your time if you give this Bill a second reading and refer it to a Committee. Nothing will result from that except delay; so why do it? The two Bills, as I have said already, are absolutely irreconcilable, as your Lordships can see for yourselves if you only compare

**Nurses' Registration in the House of Lords**—(*cont.*).

them. On the grounds of expediency alone you are justified in saying to the promoters of the Bill, "No, this may be a very good plan, but it is not the plan which is before us, which has been before us for a great many years. This is not the plan which we have decided to adopt in principle. It is not the plan in discussing which we have already in Parliament spent a good deal of time, and therefore we are very sorry, but we cannot switch off and take up another plan at the present time." So much for the reason of expediency.

Now let me turn to that which is far more important, and that is the matter of principle. But let me premise that registration is, in its essence, a very simple matter, and on that ground, and on that ground alone, it is a very grave mistake to complicate it with a totally different issue. The College of Nursing, which is promoting the Bill that is now before your Lordships, cares only about incorporation. That is the one main, primary object of their Bill. They want to get rid of the word "Limited" after their title. For some reason or another it is extremely irksome to them. They can do that without your assistance. They can do it through the Board of Trade under the Companies Act, 1867. They do not want an Act of Parliament for it, and there is no need whatever for them to take up the time of your Lordships about it.

It is just as well to be guided by precedent when you undertake legislation of this kind, and for the registration of nurses we have well-defined precedents in the Medical Acts and the Midwives Act. It is desirable to follow these precedents in order that the legislation of State Registration may be harmonised with the other great measure which is before Parliament—namely, the Ministry of Health Bill. The normal functions of a statutory governing body are education, registration and discipline; and, as my noble friend who moved the second reading of the Bill has said—he made it rather a boast, though I cannot think why—the College of Nursing does not confine itself to these three ordinary and accepted functions. It goes far outside of them into all kinds of philanthropic enterprises. Again I ask, why should you tack philanthropic enterprises on to a perfectly simple and necessary measure for the registration of trained nurses? This College of Nursing aims at all kinds of things like homes, clubs, and provincial centres. I say it is most unwise to tack such enterprises, which, after all, are private enterprises, on to a Bill of this kind.

Now let me remind your Lordships what the precedents are. You have, first of all, the great body of Medical Acts, and the medical profession has as its statutory governing body the General Medical Council. Then you have the Midwives Act, and because midwives are already registered they have their body similar to that which the advocates of State Registration wish to set up under the Central Midwives Board. Those two bodies define the standards of education; they do not educate. Their function is to supervise and inspect, and it is obviously wrong and inconvenient that those who supervise and inspect should actually be the teachers. This is one of the fundamental differences of principle between us. The College of Nursing wish to educate and have control of the education, whereas our principle is to stick to the established precedent of the Medical Acts and the Midwives Act, and confine the functions of the statutory governing body to education, registration, and discipline.

I am not in the confidence of the Government and have no notion as to what line they will take about this Bill, but so far as I am able to judge I believe, if your Lordships accept the Bill which is now before the House of Commons, you will find you are passing into law a measure which will harmonise and conform to the general scheme of the Ministry of Health Bill, and you will be doing that which will be convenient to the President of the Local Government Board in that great and important measure which he is now bringing through Parliament.

The next question of principle is the claim put forward by the advocates of this Bill, that it is more democratic than ours. I am sure your Lordships will not be misled by a question-begging use of this term, which of all political terms is most frequently misused and abused. No doubt that kind of thing is good enough for a very large section of the public, but in Parliament and in connection with a measure of this kind it is veritable clap-trap to talk of democratic principle. To talk of democratic principle in connection with the State Registration of Nurses is just as much out of place as to talk of it in connection with the Army, the Navy, with universities, schools, or colleges. It is just as much out of place as it would be to talk of it in connection with the nursery.

The point on which the whole controversy hinges is this: the College of Nursing secured a good deal of support for their Bill by loudly advertising the democratic principle which they said resided in a certain clause embodied in the seventh draft of their Bill. The Bill before your Lordships is the eighth draft, and it shows how many hesitations and doubts they have had about this measure. The clause in question, Clause 5, subsection (1) (*a*), was as follows:—

"Of the persons elected by nurses on the General Register to represent England, Wales, Scotland and Ireland, respectively, five-sixths shall be nurses on the General Register."

You will hardly believe it, but that clause, on which they base their whole claim of democratic concession, has actually been deleted from the Bill, and the Bill now lays down that the direct representatives of the nurses on the General Council shall not be "nurses," but "persons." Persons may be anybody. They may be women, or they may be men; they may be nurses, or they may be doctors. They may be the lay managers of hospitals, and it therefore by no means secures that the representatives of the nurses should be elected from the body of trained nurses. As it is, not one single seat is absolutely secured to nurses on their own governing body. The compromise which was embodied in that clause in the former draft of the Bill, on which a large support was secured and which has brought about this flood of literature that has been showered upon us, has been broken, and all this pretence of democratic principle falls to the ground.

The Bill of the Central Committee, before the House of Commons, provides that nursing opinion shall be directly represented on the governing body by the nurses themselves, and not by "persons"—not by persons, male or female, some of whom might just be those persons from whose autocratic tyranny the nurses wish to escape. If you want democratic principle, do not give preferential treatment to any one society, either to the College of Nursing, Limited, or any other society, which you would do by this Bill; and do not grant to any particular class of nurses any abatement or exemption from those fees which are necessary for conducting examinations and compiling and maintaining a Register. Charge all of them the same fee; charge them what it is necessary in order to carry out that work. They can afford and are willing to pay it. A guinea will not do it, but the nurses are perfectly ready and capable of paying three guineas, which is the charge in the other Bill.

I saw in the *Times* the other day an article which I thought most unfair. In this article the following statement appeared: ". . . a new spirit which animates nurses, and demands expression in democratic institutions." Those of your Lordships who are acquainted with hospitals and with trained nurses and other nurses, for whom we have the most profound admiration and towards whom at the present time every one has reason to feel the greatest gratitude, will certainly agree with me when I say that one sees no sign of any such new spirit. There is no doubt whatever who wrote the article in the *Times*. There can be no doubt that it is the principal promoter of this College of Nursing Bill, which provides for a monopoly for the College of Nursing and for special privileges for the members of the College of Nursing. And to put forward a claim that this Bill is to meet this "new spirit which

**Nurses' Registration in the House of Lords**—(*cont.*).

demands democratic institutions" is, if I may use a slang expression, "about the limit." I ask your Lordships, Is the present the right time or a prudent occasion to set up any kind of monopoly or create any new vested interests?

I can give you the broad distinction between the principle of these two Bills. The object of the State Registration Bill now in the House of Commons is to raise the vocation of nursing to the standard of a profession—to make it a self-supporting and self-reliant profession, of which every one of its members will be proud, and which will have the same rights and the same powers as other professions, some of which cannot be regarded as so high in the scale as that of trained nurses. I refer to midwives, whose training is far less serious than that of general nurses. On the other hand, you have the advocates of this Bill, who wish to keep nursing as a philanthropic concern, a charity, an institution to be supported by flag days, fêtes, bazaars, and by all those adventitious aids by which charities are supported. Nurses do not want that, and it is exactly from that state of things that they wish to liberate themselves.

Trained nurses want to be registered in order that their qualifications may have due public recognition, and they do not want to be compelled to become members of the College of Nursing. Under this Bill the College of Nursing says they are entitled to become members. Any one who has any knowledge of the world at all, and who can read between the lines, can see that "entitled" in this case means that very strong pressure will be put upon them to belong to the College, and the College will thus have the power to exact from them any fee or subscription that they decide to charge, and thus you will destroy all existing societies and associations of nurses. If you want to nationalise all these associations—it is possible that you do, as nationalisation is the craze at the present time—do it in the right way. Set up a Royal Commission and make the advocates of the College of Nursing Bill the judges in their own cause. Give them every opportunity of bullying and browbeating the supporters of State Registration. That is the right way. But do not do it by a side wind in a Bill which wrongly purports to be a Registration Bill.

The finance of this College Bill is utterly unsound. I hope your Lordships will take it from me that, in the opinion of those who have made a very careful estimate, a guinea fee will not pay for registration and examination. The fees to be charged should be defined in the Act, as they are in the Bill before the House of Commons, and should not be left to the discretion of those people who later on will draw up the rules. So much for the question of principle.

I come, finally, to my assertion that this is a Bad Bill. I should like to take your Lordships through the clauses of the Bill, but I have occupied so much time already, and I do not think this is the occasion. I hope that there will not be any future occasion on which it will be necessary to do so, and that this will be the last we shall hear of this Bill, because I am quite certain that if your Lordships should carry it any further you will inevitably recognise later that you have made a bad mistake. Just look at the Bill. It professes to be a Bill for the Registration of Nurses, but it starts straight off with a clause saying that the College of Nursing, Limited, is to be entitled to bear the title of "the College of Nursing," and then it goes on to say that this shall be administered by the General Nursing Council. You are not told what the General Nursing Council is. Here you are suddenly referred to a body which is not in existence and for which no provision has been made.

I speak subject to correction, but I believe the usual course is first to state in a Bill that there shall be such and such a body; next to state how it is to be constituted, and then to lay down its duties and functions. This Bill goes the other way about, and speaks of a General Nursing Council, which does not exist and for which you look in vain throughout the Bill in order to find out how it is to be constituted and what it is to be like when constituted. My noble friend did give you an explanation. It is not Parliament who is to settle how it is to be constituted, but it is to be done in this way: A Provisional Nursing Council is to prepare the scheme, and that scheme is to be approved by the Privy Council, and not by Parliament. What a roundabout way of doing things! The Provisional Council is to form the scheme, and then it goes to the Privy Council. Why not do it in the Bill, as you intended to do in the Bill which your Lordships passed ten or eleven years ago? I will not trouble your Lordships at this stage with the actual clauses of the Bill. I hope that I have said enough to show you how totally different this Bill is in principle from the Bill which only purports to be for the registration of nurses.

There is, however, one point which was made by my noble friend to which I must refer before concluding. He said that he was at a loss to understand the statement that his Bill would result in the disfranchisement of nurses. I should like to explain that to him. This Bill provides that as soon as 30,000 nurses are registered the rules are to be made and the Council is to be formed. Do your Lordships not see what will happen? There are between 70,000 and 80,000 trained nurses in this country. The College of Nursing have 14,000 already. Within a few weeks they can get another 16,000 and make up the 30,000, and then, having got the electorate entirely under their control, they will be able to settle everything. The other 40,000 or 50,000 nurses will not have a say; the mere exigencies of time and distance would prevent them. These other nurses, scattered far and wide, are very busy people, and in the ordinary course would not hear of this thing until long after the College had got 30,000 nurses. The effect would be that you would have a gerrymandered constituency which would carry out exactly those things which the College of Nursing, Ltd., wish to have.

Is it worth while to confuse and complicate the issue in this manner and to put ourselves at cross purposes with the House of Commons, who have already made good progress with the proper Nurses' Registration Bill? Is it right to delay the registration of nurses at the present time, when it has been before Parliament for so many years? Nurses have deserved well of the State and have rendered services during the past five years for which every heart in this country is full of gratitude. They stand higher in public estimation as a class and a profession than ever before. They are asking you for this thing, and is it justifiable, for any reason whatever, to delay the grant of it? Surely there is enough unrest and discontent among all classes of workers without adding to it gratuitously by passing the measure which is before your Lordships. Nurses for a long time have been grossly and scandalously underpaid, and have been subject to a great deal of tyranny and oppression. Now is the time to remedy that. Here is a thing which you can remedy at once, and the means of remedying it is the Bill before the House of Commons.

The Bill now before your Lordships is a Bill to aggravate those evils of which I have just spoken; simply for this reason, that you will put nurses more into the hands of the lay managers of hospitals than they are now. The Bill before the House of Commons holds the field, and I beg your Lordships to give it a fair field and no favour. Let it come as it is to this House, and if the College of Nursing, Ltd., so desire, let them introduce amendments, so that your Lordships can test them in the regular way. Do not put the thing into other hands by handing it over to a Committee. It is a matter which you should settle in the whole House, and not relegate to any Committee. It is a thing which your Lordships should be proud to settle in the whole House; and in legislation of this kind it is wise and safe to adhere to established precedents, and make your laws uniform and in conformity with Acts of a kindred nature. You have those Acts in the Medical Acts and the Midwives Act. I hope I have said enough to convince your Lordships that the right and fair course is to refuse a Second Reading to this Bill.

**Nurses' Registration in the House of Lords**—(*cont.*).

Amendment moved:

Leave out (" now ") and insert at the end of the motion (" this day six months ").—(*Lord Ampthill.*)

**Viscount Knutsford:** My Lords, both the noble Viscount who moved the Second Reading of this Bill and the noble Lord who moved its rejection said that registration of nurses was useful to protect the public and to protect nurses. If your Lordships will listen to me for ten minutes I will show you why registration of nurses will not protect the public and will not protect nurses. In the first place, what sort of nurses do the public want protection against? They do not want protection against nurses who are working in the hospitals. They do not want protection against nurses whose skill and character are guaranteed by the hospitals who send them out. They do not want protection against nurses sent out by institutions. They do not want protection against district nurses, against village nurses, against cottage nurses who are known to and who are working under a Committee responsible for them. Then whom do we want protection against? I suppose the answer would be, " We want protection against nurses who are working on their own." But even of that limited number you do not want protection against any nurse who is engaged by a doctor.

Therefore we come down to this, that the only people we want protection against are nurses who are working on their own and are not engaged by a doctor. The doctor almost always engages the nurse who attends to his patient. Hardly any of us, I should imagine, would be so silly as to engage a nurse to look after ourselves or after anyone we loved, simply because her name was on a Register. Why, we should not engage a cook simply because her name was on a Register. Therefore, then, for that limited class registration is not wanted as a protection for the public. Even if you do take one of those trained nurses not engaged by a doctor, what do you get? What security do you get from the fact that a nurse is on a Register? " I shall get a good nurse," is what the advocates of registration say; " if I engage a nurse from a Register I shall get a fit and proper person to nurse me." Is that so? What does registration mean? It means that perhaps ten years, or five, or twenty years ago a nurse passed a certain necessarily easy examination, and that then she was a woman of good character. But, my Lords, character may and does change, and certainly nursing changes.

Not long ago, if I may allude to myself for a moment, I had to revise the manual for Army male nurses, and every page of that book had to have some alteration made to it. Nursing changes, I will not say every day, but certainly every two years. I do not know whether any of your Lordships have had an operation, but if you have you would know that the preparations for an operation have completely changed. We used to have to put people for twenty-four hours under a wet compress. Such a thing is unknown now. The treatment of bi-antiseptics is changed. The treatment of septic wounds is entirely changed. Appendicitis was not invented till within the memory of many of your Lordships, and even now the method of nursing it is altering. Therefore, when you get a registered nurse you are not certain that your get a technically fit woman to nurse you.

All hospitals know quite well that the passing of an examination—and that is the only test you can get from registration—does not give you the guarantee that you require. You cannot by examination test what is a far more important part of a nurse—her character. You can only test that she has passed this wretchedly easy examination. But all hospitals know that the passing of an examination is not the test. All hospital matrons will tell you that it is not at all certain that women who have passed the best examinations make the best nurses, nor will the passing of an examination be a reason for any hospital giving a woman a responsible post in that hospital. You may not believe me, but here are the words of one who is dead. Hear what Florence Nightingale said upon this matter. I have a letter from her in my hands. She wrote:

" That woman's work must be the sum of individual effort is, I am sure, tremendously true. We can no more stamp a nurse by a general register or a certificate than we can a sculptor or painter or an architect; indeed much less. For these have to do with dead clay, or canvass, or brick and stone, while the nurse has to do with the living body and even mind of the patient. The idea of the new-fangled people seems to be to put nurses on the level of dictionaries —a dictionary can answer questions. . . . Hospitals do not take their own nurses from among those who are known chiefly as having well passed a theoretical examination."

She went on to say:

" When we consider the teaching of our great Master—how perpetually He dwells upon this, that it is not knowing doctrine but bearing fruit that he desires of us, and that the former is nothing in his eyes compared with the latter, which is so eminently true with regard to our nursing profession—we may well be surprised at the confusion that should have arisen between real training and theoretical examination."

Therefore what we get by the registration of nurses, which is simply the knowledge that a woman has passed an examination, is no protection to the public at all.

Nor, my Lords, will it protect the nurses. The experience of this country is that registration is perfectly useless unless there is combined with it a penal enactment that nobody is to nurse or is to practice unless registered. I happen to be on a Government Committee inquiring into the Dentists Act. There is here a good example. The dentists had voluntary registration, but because there was nothing to prevent men who were not registered dentists from practising as dentists the whole country to-day is flooded with men with no dental qualifications at all, and they are practising, and practising more successfully than the registered dentists, because they are not bound by the same rules. They are not allowed to call themselves " dentists" any more than these unregistered nurses will be allowed to call themselves " registered nurses," but they are able to put on their doors " Dental surgery " or " Dental surgeon," just as these women who are not registered nurses will be able to put over their doors " Nurse."

Therefore this registration of nurses is no protection at all to the nurses themselves. You cannot make it penal; you cannot say that every woman who is not a registered nurse shall not be allowed to nurse, because if you did that you would stamp out some of the very best work that is being done in the whole country. You would stamp out every district nurse because they happen not to have had the full hospital training, though they are able to do God's own work in every village they live in. You would stamp out cottage nurses, you would stamp out village nurses, you would prevent the V.A.D.s—and, if I may say so with gratitude, we must all recognise the work that Lady Ampthill has done in connection with the V.A.D.s——

Several Noble Lords: Hear, hear.

**Viscount Knutsford:** If you made it penal to nurse unless a woman is a registered nurse, then no V.A.D. could ever do any bit of nursing that she felt inclined to do. They do not claim to be nurses, but within a limited sphere these women who have not had full hospital training —and I plead for those women, too—are all able to do immensely useful work. And therefore you cannot pass a law which prevents people nursing unless they are registered nurses; and unless you do that you make all registration perfectly useless.

Lord Ampthill has wondered why he finds himself going to vote against a Bill for the registration of nurses. After what I have said, it is perhaps a greater wonder that I find myself an advocate for a Bill which is going to pass registration of nurses, because I am certainly going to vote for the Bill introduced by Lord Goschen. I will tell you why. I feel that I am now a voice crying in the wilderness, though I believe the voice was right; that I have no longer the support for my views (which I know

**Nurses' Registration in the House of Lords**—(*cont.*).

are right), and therefore I feel that I must take the best course that I can, and, without admitting that I was wrong, I must do my best to rectify what you may think my mistake.

But I am quite certain of one thing, and I speak with some knowledge. I am quite certain that Lord Goschen's Bill is by far the better of the two; I am quite certain that it carries behind it the opinions of all the best workers in the hospital world. Only the other day there was a very large meeting of matrons representing all the hospitals in London except one, and provincial matrons, too, and it was unanimous in favour of this Bill and against the other one; lately there has been a meeting of all the hospitals, again unanimously in favour of this Bill. I think we are under a very great disadvantage here, because we are obviously unable to discuss the clauses of a Bill which is not before us. But I am really not so frightened as Lord Ampthill is (if I may apply the word "fear" to him who has never shown it in any other way). I am not so terrified of differing from the House of Commons. They have had their Bill; may not this House have its own Bill too? Why should not we have a better Bill than the House of Commons Bill? And certainly if you could take the votes of the nurses you would find that this Bill was supported by them, and not the other.

There has never been an organisation to which so many nurses have belonged as the College of Nursing, and is it to be thrown in the face of the College of Nursing that it is helping nurses in many other ways than registration? Lord Ampthill said something about the tyranny over nurses. Why, the Bill in the House of Commons will not alter that tyranny. His other boast, almost in the same breath, was that all that the other Bill did was to go in for the register of nurses and nothing else. How can it touch the tyranny of nurses? Whereas this Bill does do something; it does set up this College of Nursing, which has high and better ideals for nurses.

I am quite convinced—and that is what makes me vote for this Bill against all the principles I have held so long—that this Bill will be for the benefit of nursing. I do hope that your Lordships will give it a Second Reading, and when it is given a Second Reading—it may cause delay (I do not doubt that, but we cannot help it)—do let us try and get from the representative of the Local Government Board, whom I see here, some assurance that, whatever differences there are between the two Bills, whatever councils are set up, shall be made the subject of an inquiry or a Committee. You will never agree; do not think there will ever be an agreement. I know enough of the nursing world to know that, after twenty-three years. It is as much as one's life is worth to go through the Lobby now. You cannot get an agreement. But you may get a Council forced upon them which will carry the confidence of nurses after the first sore feelings have passed off. For what my opinion is worth, I beg you to give this Bill a Second Reading.

**The Marquess of Crewe**: My Lords, I should not have taken any part in this debate merely because I happen to sit on this Bench, but it so happens that this subject is one with which some years ago I was closely concerned. At the time mentioned by my noble friend below the gangway, Lord Ampthill, eleven or twelve years ago, when this was a matter of discussion, I filled the office of President of the Council, and was consequently the Minister concerned with this subject. I am only sorry that the noble Earl who leads the House is not able to be here (although I am certain that his place will be well filled by whatever Minister deals with this question) because it is one of the comparatively few matters on which he would have been able to enlighten us as a departmental Minister, apart from the great public work in other offices which he now undertakes.

I very well remember how high feeling ran at that time, and we have full evidence that it runs equally high now. There are marked divergences of opinion even between those who would welcome the establishment of a Register of Nurses. My noble friend who has just sat down has declared himself a very reluctant convert to the necessity of such a Register, though he has not changed his opinion that it will be in effect a useless addition to our political apparatus. I well remember in the former discussion that the arguments which were used by Lord Knutsford were freely advanced, and I do not know that there is any complete answer to them. It is quite true, as it appears to me, that the mere fact that a nurse has passed an examination some time before and that her name is on the Register is not in itself a guarantee either of her character or her efficiency.

It is sometimes said, "You are content to place confidence in a Medical Register; why not, therefore, be content to do the same with a Register of Nurses?" But, as your Lordships will at once see, there is no real analogy between the two professions. A doctor has to work perpetually in the light of day. The mere fact of his being on the Medical Register will not save him or cause his practice to extend if people see that he is inefficient, if funerals are almost the invariable sequel to his series of visits, or if he displays signs of some kind of physical incapacity, conceivably the result of intemperance. But in the case of a nurse there is no such safeguard. She is sent for from her home or from some institution, and it is entirely on the character of the institution from which she comes that you rely, because her work is not done in public in the sense which that of a doctor is. Therefore I well remember that it was always hoped that when a register was instituted it might be possible to supplement it by some kind of continuous renewal of a certificate at stated intervals. Whether such a plan would be in fact feasible I do not pretend to say, but if your Register is designed to be a real guarantee of efficiency, or of the newly acquired efficiency which Lord Knutsford points out is so requisite in nursing, it will be necessary to devise something of the kind.

One point of view has always been that the nursing profession cannot be regarded as a quite independent profession in itself, but that the nurse must be always regarded as a kind of *aide-de-camp*, as it were, to the doctor, and that therefore the medical profession ought to exercise, if not a predominant influence, at any rate a very great influence in any body framed to conduct the affairs of the nursing profession. On the other hand, there are many in the nursing profession who themselves claim the most absolute and complete independence for their profession—so honourable and distinguished as we all know it is—and resent the idea that outsiders, even such closely allied and distinguished outsiders as the medical profession, should take much part, or indeed in some cases any part at all, in the management of the profession. Those differences of opinion have not been passed. But now there is a certain novelty in the situation—a definite novelty since my time—by the establishment of the Royal College of Nursing. Those who, like myself, have not been able to keep up any continuous knowledge of this question have very little acquaintance with the work, with the standing, and with the hopes of the Royal College of Nursing. When, therefore, we are told—as Lord Ampthill has told us—that this body is claiming by its Bill a paramount place in deciding the future of the whole College of Nursing, I confess that without more knowledge I am not able either to support or to dispute that plea.

We are in this further difficulty, that we have not got before us yet the other Bill, which I suppose ought to reach us soon after Whitsuntide, and to pronounce an opinion either for or against this Bill without having seen the other appears to me an almost impossible course to take. On the whole, therefore, it appears to me that the wisest course for us to take is that which has been favoured by my noble friend who has just sat down—namely, that this Bill should receive a Second Reading,

**Nurses' Registration in the House of Lords**—(*cont.*).

but a Second Reading which does not imply any recognition of its superiority (if it is superior) to the other measure.

The noble Viscount, Lord Goschen, appears to have been almost accused of what is known as "jumping a claim" in having introduced this measure before the other Bill has reached us. I think we ought to regard this one altogether without prejudice until we have seen the other. When the other comes, if your Lordships are so impressed by its superiority over this measure it could be proceeded with; or if we are not able, after it has been read a second time, to decide that it is definitely superior to this, then I trust that His Majesty's Government will recommend the course of sending both to a Select Committee. I do not think that any serious waste of time need be involved in that course—that is to say, not such waste of time as will prevent the chosen Bill, the ultimate Bill, from becoming law this year. That we all hope some such measure will do. We know very well—and my noble friend here (Lord Knutsford) recognises the fact—that the establishment of a Register is strongly demanded by the vast majority of the nurses themselves. I think that is quite enough for us. That being so, we ought not to delay any longer than can possibly be helped. But it clearly will be idle to proceed with this Bill in any way until we have seen the other; therefore I hope that His Majesty's Government will adopt the course I venture to support.

**The Earl of Mayo:** My Lords, after the speech to which we have just listened from the noble Marquess it is quite a "toss up" now which Bill the House will accept. It is not for me, however, to make any further remarks about that. Referring to the speech of Lord Knutsford I feel very diffident indeed in the face of his experience of nursing and hospitals about saying anything, but I understood from that speech that the noble Viscount does not want any registration at all. I also understand, however, that the nurses throughout the United Kingdom want registration; and I must point out that the midwives, who are really most important nurses (as I know, Lord Knutsford will admit) are already registered. I believe the noble Lord (Viscount Knutsford) is going to vote for Lord Goschen's Bill. It is a sort of "Hobson's choice," and he thinks that on the whole it is the better Bill.

Lord Goschen said that the whole matter had been much too long delayed. I agree. I think I spoke in 1908, when there was a Bill introduced—which I must remind your Lordships passed its Second Reading without a Division in this House—with regard to the registration of Nurses. The matter has been too long delayed, yet the noble Viscount introduces a Bill on the top of the Bill that has passed its Second Reading in the House of Commons, and gone through the Committee stage; and the effect of the introduction of this Bill will be to delay the matter very much. The noble Viscount says his measure has the spirit of democracy in it. I must point out that what nurses want on the Council are not persons to represent them; they want personal representation themselves. That is the point which they make above all things, and I think that that matter should be considered by this House, and very seriously considered. After all, nurses are a body for whom we have the greatest respect. During this war many have sacrificed their lives, and we owe a great deal to the nurses.

With regard to the Nursing Council, which is mentioned in Viscount Goschen's Bill, it is quite true that those who govern the nurses on the Council might be the employers of the nurses. That is a thing to be avoided very much indeed, but this is not the time to criticise the different clauses in the Bill. I should like to support Lord Ampthill on these grounds—that there is a Bill before the House of Commons now, and that Bill is likely to come up to us, as the noble Marquess said, after Whitsuntide. Why should the whole issue be confused by another Bill? I think it is much better that the House should consider the House of Commons Bill and not vote for that of the noble Viscount. A very good remark was made with regard to the College of Nursing, Limited, as I think it is called, at a meeting held to protest against Lord Goschen's Bill. It tickled my fancy very much. It was a remark made by Dr. Couch, who said he compared the College of Nursing to Rostand's "Chantecler," which, when it crowed, was under the impression that it made the sun rise.

**The Marquess of Dufferin and Ava:** My Lords, in addressing your Lordships' House for the first time I do not propose to occupy your attention for long, and, in any case, I have great diffidence in speaking on this very thorny and difficult question, which really almost requires the experience of a life-time thoroughly to understand. Your Lordships have already been reminded how, in 1908, we passed the other Bill, and it seems to me that, if you support the Second Reading of the Bill now before the House, you will be forsaking the old love for a new one, and, if I may say so, the old love is far and away the more attractive. We, who oppose the Bill of the College of Nursing, do so on several grounds, but I think one which may commend itself to your Lordships is—if what I maintain is true—that the Bill before your Lordships at present is a hospital governors' and matrons' Bill, whereas our Bill is a Bill of the rank and file. Among those who composed the Bill there was not, I believe, one single working nurse. The Council is composed of matrons, hospital governors, and medical practitioners.

The College of Nursing has made great capital out of the fact that it has 14,000 members. I do not think this is the least surprising if you see an extract from a circular, widely distributed all over the United Kingdom, in which the nurses were told that if they joined the College of Nursing they would at once get on the Register without further expense and without examination. So, of course, naturally they all joined the College of Nursing, and I do not think this matter of numbers should weigh with your Lordships at all. Why so many nurses distrust this College of Nursing Bill is because the College is so obviously working for its own hand. It is composed of people who for years have strenuously opposed the registration of nurses, and I think it is not unfair to suggest that what was in their minds was that they should produce their own Bill with clauses which still kept the nurses under their domination The chief reason why hospital governors and matrons were always opposed to registration was that the standardisation by the State of the qualification of nurses would tend to make nurses independent of the Hospital School by reducing the great amount of competition from unskilled nursing workers, which would tend to raise the salaries not only outside the hospitals but in the hospitals themselves. Registration really meant the abolition of cheap nursing labour.

Your Lordships may have noticed that the Press are rather inclined to support the Bill which is now before the House rather than the other Bill, but I think that you should not attach too much importance to this, because the so-called Nation's Fund for Nurses, which was raised to endow the College of Nursing, spent a very considerable sum on advertising and, without throwing any doubt on the genuineness of the convictions of the Press, I think that perhaps the sentiment, "You scratch my back and I will scratch yours," may have reacted a little between the Press and the College of Nursing. One very distinguished supporter of the Bill now before your Lordships' House was heard the other day to boast that he had got Lord Northcliffe. I do not know if that was an idle boast or not, but in any case I do not see that we should be unduly depressed by it, because though he may have got Lord Northcliffe to-day there is no certainty that he will have got him to-morrow. But do let us have the better Bill. Do not let any of us vote for the Second Reading of this Bill unless and until he has compared it with the other Bill. Your decision this evening affects a great number of people of whom we are all very fond and to whom we owe a debt of gratitude. Personally I feel so much indebted to them that I intend, in however humble a way,

**Nurses' Registration in the House of Lords**—(*cont.*).

to do what I can to forward what I hope are their best interests.

**Lord Greville**: My Lords, I hope that your Lordships will give a Second Reading to this Bill. We have listened to several speeches this afternoon, and I think every speaker has admitted that the registration of nurses is much called for and agreed upon in the nursing profession. After listening to the speeches for and against what I am personally convinced of is that there is a very large measure of disagreement as to the method by which it should be carried out, and I think it would be extremely difficult for your Lordships' House to agree to any Bill which has been introduced in another place and which you have not seen. Therefore, I hope that you will give this Bill a Second Reading, and later on, when opportunity offers, put both Bills in Committee, thereby securing an agreed Bill and the better Bill of the two. The argument used against this Bill by the noble Lord, that he was the first in the field, does not move me much. You may be first in the field, and yet not produce the best Bill. He also said this Bill ought not to be introduced because of the Bill which is already in the House of Commons. I do not think that argument carries great weight. Your Lordships' House had always prided itself upon its fairness, and with these two Bills in their present state it is only fair to give this Bill a Second Reading, and then form your judgment later on when you have an opportunity of seeing both Bills.

**Earl Russell**: My Lords, I have always been in favour of the registration of nurses on grounds which affect us all, because we recognise it raises their natural *status* and gives security to the public. When I saw that this House was going to be invited to read a second time a Nurses' Registration Bill I was naturally pleased and gratified. I confess, when I learned that the Bill was likely to be supported by Lord Knutsford, I developed what I think is not altogether an unreasonable bias. The noble Lord has told us that he has always been opposed to the registration of nurses altogether, and when I find a reform introduced in two forms (*a*) and (*b*) and the person who has always been most bitterly opposed to that change supports one, I confess I feel it is all the more certain that the other form is the most effective, because he will support the one which it is likely will go the less way to carry out that which he does not like. That appears to be ordinary common sense.

Your Lordships, I think, are aware probably that the other Bill has progressed a considerable way in another place; it has, in fact, passed the Standing Committee. In the Standing Committee, in some of the proceedings that I saw reported, it appeared that Amendments had been proposed and moved on behalf of the Government and incorporated in the Bill, and that certain compromise Amendments had been assented to. If that is so it is rather peculiar, when that Bill is almost on the threshold of your Lordships' House, we should find this body introducing here, with a view of confusing the issue, a Bill which proposes the registration of nurses in a totally different way.

I am a believer in the principle of self-government, and I am quite prepared to take up the noble Viscount on his statement, which certainly astonished me, that the majority of nurses are in favour of his Bill. If I was satisfied that that was so I should be prepared to support the Bill. But, I confess, that neither my post-bag nor the appearance of the Lobby tend altogether to support that view. If it is correct, the supporters of Lord Goschen's Bill are certainly very retiring and silent as compared with the supporters of the other measure. What evidence is there to be produced, or that has been produced, that the majority of nurses favour the Bill now before your Lordships' House? I see no signs of it; and it is an ingenious way of getting the thing up.

The position is this. Women now have the vote; they have to be considered more than they used to be. It is not so easy to say, "I do not like the registration of nurses." Women are now likely to get what they insist on having, and the noble Lord tells us that he recognises they must have registration. But, he says, "let me see how I can make it as little harmful as possible." The College of Nursing, Limited, does not attract the nurses by the force of its own virtue or its past history (which is a short one) but it attracts them by the remarkable circular to which your Lordships' attention has already been called, and of which, I think, it is worth while reading the exact words—

"Every certificated nurse should apply at once for registration by the College of Nursing. (1) Because the Council of the College of Nursing has drafted a Nurses Registration Bill which provides that the Register already formed by the College of Nursing shall be the first Register under the Act. If, therefore, you are on the College Register you will automatically and without further fee be placed upon the State Register, when the Nurses' Registration Bill is passed."

That sort of statement does not take in your Lordships, or any lawyer, or anybody with Parliamentary experience. But what does a nurse think when she receives a statement like that? She naturally takes it at its face value, and she probably takes it for granted that it is a proper and natural way of getting on the Register, and that if she wishes to be in her proper place she should get on the Register of this College of Nursing as soon as possible. In that way you get a large membership, and you come to this House and say, "This should be the dominant body because it has the largest membership."

I am not prepared to analyse in any detail, and I think it would be out of order to do so, the exact method of election to the Council as between the two Bills. I think the Council which gives nurses direct representation—which gives them the representation for which they ask and insist—is in the Bill which is not before your Lordships' House. I think your Lordships would be unwise to confuse the issue by giving a Second Reading to this Bill. The noble Marquess said that we could give a Second Reading to the Bill without assenting to the principle of the measure; but if we went to a division it is difficult to say we have not assented to the principle, and I think you would have prejudiced the issue in an unfortunate way. If this is the proper way to constitute the governing body of nurses it will be perfectly easy to do so by an Amendment to the other Bill when it comes before us.

Notwithstanding the statement of Lord Knutsford, that there is a majority of nursing opinion in favour of this Bill, there is not the slightest evidence of it. The opinion I have formed from all the literature I have read—and I have taken some pains to inform myself on the subject—is that the overwhelming mass of nurses are opposed tooth and nail to this Bill, and even if it were to become law—though I am convinced it would not pass the other House—you would find an unofficial Register compiled in opposition to it. I hope that your Lordships will hesitate to give a Second Reading to a Bill which is merely intended to camouflage the real issue.

**Lord Buckmaster**: My Lords, I cannot help thinking that it is unfortunate that we are called upon to decide a matter which each one of us desires to settle for the best possible interests of the nurses (and of them alone) in a manner which prevents us from really being able to comprehend what are the issues that are involved. No doubt your Lordships, in common with myself, have received during the past fortnight a considerable quantity of literature, and I have no doubt you have done your best, as I have, by reading it, to try and comprehend what is the real matter upon which we are asked to make up our mind. I must admit I have come to the conclusion that it is quite impossible to determine that issue by anything in the nature of a debate in full assembly of this House. There is to my mind only one possible way in which it can be settled, and that is by having the whole matter thrashed out before a Committee of the House. If that is right—I cannot help hoping and thinking that the speeches your Lordships have heard to-night must have impressed you with the fact that it is right—is there any reason why the Bill which has been

**Nurses' Registration in the House of Lords**—(*cont.*).

introduced should be excluded from consideration before that Committee? I think there is not, and I will tell you why.

It seems to me that the real difference between the two Bills is to be found in this—that the Bill which Lord Goschen has introduced has associated with registration something beyond—something that will provide for the education and for the improvement of the standing of nurses; whilst the other Bill aims merely at blank registration, which, as has been pointed out by the noble Viscount, Lord Knutsford, is in itself an ineffectual thing. Lord Knutsford, as I understand him, supports Lord Goschen's Bill not for the reason given by Lord Russell, that he hoped by this means to get rid of registration altogether, but because he hopes by this means to make registration a reality and something that is of value and not something which is nothing but a name. Registration by itself is nothing but the compiling of a dictionary. There are registered plumbers; there are registered auctioneers, and all kinds of people are registered, and registration by itslf conveys neither authority, nor skill, nor character of any kind, nothing but the compilation of a book. If you want to make registration real you must associate with it something that will establish the *status* and improve the position of the people who register. Whether this Bill will do that, I find myself unable to say; but this I know, that that is claimed for it and it is that which has caused Lord Knutsford, whose work in connection with hospitals we all know and respect, to support the Bill and to ask your Lordships to give it a Second Reading.

I do not desire, after the long debate that has taken place, to trouble your Lordships further with considerations of my own on this matter, but I think it would be a serious thing to reject the Bill which Lord Goschen has placed before your Lordships for consideration, and to say that it is not to be considered at all, when all he asks is that when it has been read a second time your Lordships may consider the two Bills together and out of them make the best Bill that can be compiled for improving and establishing the position of the women, to whom we all owe an indebtedness which we are only too eager to express and discharge.

**The Earl of Denbigh:** My Lords, as chairman of a well-known hospital in London I should like to associate myself with what has fallen from Lord Knutsford and also from Lord Buckmaster and other speakers on this side. I certainly think that the most practical course will be to read this Bill a second time and refer it to a Select Committee, and then see whether it is not possible to thresh out the whole thing before that Committee and get an agreed Bill.

**Viscount Sandhurst:** My Lords, it will perhaps be convenient if I very shortly state, in the first instance, what is the view of the President of the Local Government Board; and then perhaps your Lordships, as I have had something to do with such matters, will allow me to say a word or two of my own. I share with the noble Marquess, Lord Crewe, the regret at the cause which keeps my leader, the Lord President of the Council, from his seat on this occasion.

My Lords, on my own behalf, and on behalf of the President of the Local Government Board, I hope that you will consent to give a Second Reading to this Bill, on the ground that it embodies the principle of registration, for which the Government desires to secure legislative authority as a protection for the nursing profession itself and also for the public. Having said this, it is my duty to add that I am not in a position to commit the Government in any way to the bodies described, nor to the actual College so described in the Bill. As your Lordships know, there has been a controversy over this question for very many years. I think it was going strong, as we say in another place, when I first entered the hospital field as long ago as 1883, and my right hon. friend proposes to see, by conferences and conversations with the people supporting the two Bills, how far it is possible to get some agreement, or any rate to see how much of what is left of each Bill can be taken in the near future, or immediately, as the basis for a really good Bill.

A suggestion for a Select Committee has been made in two different ways. I am not quite sure whether I understood the noble Marquess correctly, but I understood him to say that he hoped we would read this Bill a second time, and that when we had got the other Bill before us it would be a good plan, if an agreement had not been reached and one Bill could not be made out of the two by various conferences, that they should then be subjected to investigation by a Select Committee. On the other hand, I rather gathered that my noble and learned friend Lord Buckmaster suggested that this Bill should be sent at once to a Select Committee.

**Lord Buckmaster:** My idea was that when the other Bill came up they should both go together to a Select Committee.

**Viscount Sandhurst:** Then I gather that my noble and learned friend is in agreement with the noble Marquess beside him. Of course, that would give us a little time for these conferences to which I have referred. I am not in a position, as my noble friend will understand, to give any undertaking on the subject at this moment, but I will convey to the President of the Local Government Board what is their wish and what I have heard expressed in the same direction from other members of the House, and I have not the smallest doubt that he will give it most careful consideration.

Now I should like, having expressed what I may term the official view of this Bill, to say a word upon the general question. I do not refer to the other Bill, because first of all it is not before us. I listened with the great attention which is due to Lord Knutsford, who has had a long experience of these matters, and I do not quite take the same view of the change of mind of my noble friend as was taken by the noble Earl opposite. We all change our minds sometimes on questions of importance, and it has been my experience that those who change their minds generally become more vigorous supporters of the new view than they were of the old. I can go, however, a certain way with Lord Knutsford. I agree that registration in itself is not an absolute guarantee of the value of services of this or that profession. I do not care who it may be who attempts to define the real qualities of the nurses; to my mind they are absolutely undefinable. You can have a Register which will put down the age of a nurse, the high qualifications of a nurse, having passed this examination or that, and all the rest of it, but there are certain qualifications for a nurse which you cannot ascertain until you get her into a sick room. There is that curious electric spark of sympathy which passes between the nurse and her patient—an influence which is greater than any disciplinary form of mind. The nurse is able to exercise it not only upon her patient but upon every one in the house with whom she comes in contact. Such a quality as that it is quite impossible to define in writing or in any document official or otherwise.

But I quite agree with what was said, I think by the noble Marquess and also by my noble friend, that there is no room for any doubt whatever that the nurses as a whole—I believe almost without exception—desire that there should be some form of registration. Even if one thought that registration might not be quite as efficacious or so much a protection as is claimed for it by some, the fact that the nurses themselves desire it would, in my opinion, be a sufficient reason for supporting the general principle of registration. The noble Marquess who addressed your Lordships for the first time, and whom, I observe, your Lordships all heard with pleasure, remarked that this was regarded as a Bill for matrons and lay governors. I have been a lay governor of two or three hospitals since 1883, and I can assure the noble Marquess that I knew a great many lay governors and lay matrons who were in favour of registration as long ago as that; indeed, if I am not

**Nurses' Registration in the House of Lords**—(*cont.*).

mistaken we heard endless evidence upon the subject of State registration before a Committee of which I was chairman that inquired into the whole of metropolitan medical relief. It is quite an erroneous view that matrons and those in authority have been against registration as a whole.

It may be doubted if Parliament would agree to leave to a private body, or even to a statutory body, a dominant position as to the conditions of the first Register. That Register must necessarily include a large number of nurses of a training below the standard that will be considered satisfactory in the case of applications subsequently for admission to the Register. Look at the number of V.A.D.s. who have served the country for three or four years, and who are not qualified by examination certificates, but who have done most noble and self-sacrificing work. That work deserves consideration. There should, therefore, be inserted in the Bill some guidance as to admission to the first Register. I think it is agreed that it would be desirable to secure a number of persons of special knowledge and experience in the work of nursing training schools for service both on the Provisional and the Permanent Council. There are a variety of questions which require careful study. There is, for instance, the question of Supplementary Registers which opens up a field for controversy; there is the question of the class of nurses to be admitted; and there is the question of the *personnel* of the Provisional as well as the Permanent Council. I sincerely hope that your Lordships will give a Second Reading to the Bill so that we may ascertain by patient inquiry and conference how best we can get a measure which will meet the wishes of almost the whole body of the nursing profession and the public. I desire to support the Second Reading of the Bill.

On question, whether the word "now" shall stand part of the original motion?

**Their Lordships divided Contents, 62; Not-Contents, 22. Majority for the College Bill, 40.**

